# Hospital Meetings, &c.

**Published:** 1900-04-28

**Authors:** 


### HOSPITAL MEETINGS, &C.

### ST. GEORGE'S HOSPITAL.

Lord Cork presided at the quarterly and special Court of Governors of St. George's Hospital, held on the 20th inst., when there were present: The Earl of Cork and Orrery, K.P., Sir W. W. Karslake, Q.C., Admiral Sir George Willes, Major-General Gifford, Deputy Surgeon-General Penny, Dr. Duxa, Dr. Ogle, Dr. Rolleston, Dr. Penrose, Dr. Ewart (the senior physician), Mr. W. Haward (the senior surgeon), and Messrs. Shaw Stewart, Timothy Holmes, R. Thorp, F. E. Villiers, W. H. Bennett, H. L. Albert, H. W. Allingham, and C. T. Dent.

The minutes of the Weekly Board were read and confirmed, and the following letter, which has been sent to the editor of the *Times*, was ordered to be printed and circulated:—

"Sir,—We were directed by the Weekly Board of Governors of January 3 to request the favour of your insertion of the following letter. The annual distribution of the Prince of Wales' Hospital Fund has recently been made, and the particulars were announced in your issue for December 22. Of all the great metropolitan hospitals which applied for a share in that distribution, St. George's was the only one refused. This refusal was accompanied by the remark that its cause was the pecuniary position of the hospital, while free praise was given to its management and to the efforts on improving its internal arrangements, on which a sum considerably exceeding £20,000 is now being expended. Now this must tend to confirm an opinion, which we hear is widely entertained, that St. George's Hospital is too rich to need support; and we would ask your aid in removing such an impression, which is wholly unfounded. True it is that St. George's is not in so precarious a position as many of our metropolitan hospitals are; but the following rapid summary of its finance will enable your readers to see that it is in very real need of help. The year 1898 is, of course, the last for which complete accounts are ready, but those for 1899 tell much the same general tale. The income is made up of two main items—ordinary £25,197, and extraordinary £11,844. The expenditure divided in the same way shows—ordinary £34,452, and extraordinary £8,766. Thus the ordinary expenditure shows an excess of £9,255 over the ordinary income. And the ordinary expenditure is constantly rising as new and more efficient methods of treatment are devised, as it becomes necessary to spend more on nursing, and as the comfort of the nurses and servants is better provided for. The statistical table published in the annual report shows that for the decennium 1873 to 1883 the average expenditure was not much over £23,000, and for the next decade under £26,000, since which it has been steadily rising, and may now be taken at £35,000. It ought also to be known that, in consequence of its possessing a country branch at Wimbledon (not a mere convalescent home) to which the less acute cases are sent, St. George's has under treatment in its wards a very large proportion of cases in the acute stage of disease. The ordinary income is made up chiefly of (1) subscriptions and donations; (2) dividends on realised property and rents. It is in the first-named of these sources of income that the governors have to lament a great falling off. The average of subscriptions and donations from 1873 to 1883 was over £10,600, and in the next decade over £9,200. It is now below £8,000, and that in spite of continuous appeals and efforts to obtain new subscribers, and in spite of the wealth of the district in which the hospital stands. And there can be no doubt that the cause of the limited subscription is the general belief that the hospital has immense realised property and is in no want—a belief which the governors fear the report of the Prince of Wales's Fund will tend to confirm. It is true that St. George's has received in the last thirty years several large legacies, so that the amount received for dividends has risen from an average of under £4,000 in the decade first named to £13,662 in the year 1898. The rents remain at about the same sum (£1,600) during the last few years. But it will be seen at a glance that the increase in realised income does not balance the increase in ordinary expenditure—the former being under £10,000, and the latter about £12,000. In other words, the hospital is not now in so good a pecuniary position as in the period between 1873 and 1883, except (for the governors have no wish to overstate their case in any way) that it has a larger fund to fall back upon as it becomes necessary to sell out stock. The governors have always freely acted upon the conviction that the money bequeathed to the hospital by testators is not given to them to hoard up, and so to save the present managers the trouble of appealing for help; and therefore they have spent money liberally in the endeavour to make St. George's as perfect as a hospital at the present day can be; and the paragraph of the report of the Prince's Fund which gives their reason for not making a grant shows that in the estimation of competent judges that endeavour has not been unsuccessful. But no riches can hold out against a constant excess of expenditure over income—for legacies are very uncertain, and the same error which prevents people from subscribing prevents them also from bequeathing. Much more might be said but for the fear of wearying the reader. One word in conclusion. It must be far from the mind of the managers of the fund to injure a hospital of which the reigning Sovereign is always president, and of which His Royal Highness the Prince of Wales is a gracious and liberal supporter. Yet there can be no question that individual hospitals lose subscriptions in consequence of their friends preferring to give to the fund, and some of our former subscribers have given this as their reason for ceasing to contribute. To the other hospitals this is more than recouped by the sums allotted from the fund. But to St. George's, which receives nothing, the loss is uncompensated. The governors therefore feel it their duty to do all in their power to urge on the public the need for increased support, and trust that they may have your powerful aid in that effort.—We are, sir, your obedient servants,

"Timothy Holmes, Treasurer.
"C. L. Todd, Secretary.
On behalf of the Weekly Board of Governors."

The annual report of the Weekly Board for 1899 was then presented and adopted. In this it was stated that during the year 4,396 patients (523 of whom were under ten) were admitted. Sixteen persons were brought in dead. The daily average of patients was 323·41. The mean residence in the hospital was 25·08 days. The out-patients numbered 29,713, including 451 lying-in women delivered at their own homes and 2,296 dental cases. The ordinary income was £25,901; the ordinary expenditure, £36,800. The extraordinary income (legacies) was £4,402; the extraordinary expenditure (chiefly on account of conversion of nurses' cubicles into bedrooms, new kitchens, and other alterations and improvements to the hospital), £8,068. Annual subscriptions amounted to £5,949; donations to £1,278. The Hospital Sunday Fund contributed £2,083; the Hospital Saturday Fund £360. Considerable attention was devoted in the report to the circumstances in connection with the absence of a grant from the Prince of Wales's Fund, as set forth in the letter printed above. A duly-accredited inquiry officer or almoner has been appointed in the out-patient department. The report stated that it had been the practice of the hospital for many years to inquire into the circumstances of those who applied for relief, with good results. It was, however, thought that someone specially trained for the work would be able to prosecute these inquiries better than the clerk who had been in the habit of doing so; and in consequence a lady trained at the Royal Free Hospital was selected for the purpose. The almoner now saw the applicants, satisfied herself as to their social fitness for treatment, and also, in communication with the Charity Organisation Society, obtained assistance for those in need, and was able to send to the country some likely to benefit by residence there. She also made inquiries into the circumstances of in-patients needing surgical appliances, to ascertain how much they could contribute to the cost. The Board had been desirous of purchasing a Röntgen Ray apparatus, but had been deterred by the expense, and were, therefore, the more grateful to Mr. and Mrs. West, who had presented them with so valuable and necessary a gift. A friend of Mr. Timothy Holmes, one of the treasurers, had paid the whole expense of refurnishing the Drummond ward. The furniture at present in use in the wards was purchased many years ago, and it was greatly hoped that other friends would follow the example. A pathologist had been appointed at a salary of £75 a year, the Board considering it necessary for the proper treatment of the patients. The Rev. C. Hambleton Chrymes had been appointed to the office of assistant chaplain. Stringent rules had been introduced as to the milk supply of the hospital to prevent patients or staff contracting tubercular disease from this source. The Duke of Richmond and Gordon had accepted the office of treasurer vacant by the death of the Duke of Westminster, to whose memory a warm tribute was paid; as also to that of Mr. John T. Mould, a governor of the hospital since 1848, and for many years a frequent chairman of the board. Regret was also expressed at the loss of Lord Clifden and Major Grimston, two other governors very active in the work of the hospital. The death of Mr. G. F. Eland, who had acted as solicitor to the hospital for 34 years, was also reported with regret; the vacancy thus caused had been filled by the appointment of Mr. W. F. Nettleship, now the senior partner in the firm of which Mr. Eland was a member.

In consequence of the death of the Duke of Westminster, it became necessary to substitute the name of some other gentleman as a trustee. As on every occasion of the resignation or death of a trustee this had to be done, the board thought it desirable that the property of the hospital should in future stand in the name of "The Vice-Presidents, Treasurers, and Governors of St. George's Hospital," so as to save the expense and trouble caused by the change of trustees. At a special court of the governors held in January this was agreed to, and the board authorised to make the necessary alterations in the laws. The office of trustee had, therefore, ceased to exist, the duties formerly devolving on trustees being now undertaken by the board, and the seal of the hospital affixed to documents which formerly bore their signatures.

The report was adopted *nem. con.*, but on the motion to receive the auditors' report of receipts and payments for the year, Mr. Shaw Stewart thought it should not be passed over *sub silentio*. As a former treasurer he regretted very much the extent to which their subscriptions had dropped off and their expenditure gone up, the latter exceeding their income for last year by £15,177. If they were to continue at that rate it would end in St. George's Hospital going bankrupt. There seemed to be a race between the London hospitals as to which could spend most, and he felt that it would end in their outrunning the purse of charity, and in being taken over by the Government and the appointment of Government inspectors. He characterised the expenditure of £45,000 in one year as monstrous and extravagant. When he was treasurer he liked to get money's worth. He did not believe in treating poor people as dukes and duchesses. He hoped the £8,000 which he saw they were to be asked to vote to provide for payments to contractors would be final, and that there would be no more tinkering at the hospital. That meeting, the principal quarterly meeting of the year, at which these accounts came up for discussion, used to be held in May, and in view of the difficulty they had had that day in getting a quorum—one of their doctors having had to be fetched away from his duties, and their senior surgeon and physician being kept from their patients also—he should move that in future no quarterly court be held in Easter week.

The Chairman deprecated some expressions used by Mr. Shaw Stewart. He understood him to call attention to the fact that their expenditure was great and growing, and thought he should have pointed out where he considered there had been extravagance.

Mr. Timothy Holmes maintained that their expenditure was not extravagant. He had explained in his letter to the *Times* how it was that it increased. In no industry in the world had such great progress been made as in medical and surgical practice. He believed that all their expenditure was justified and was remunerative, and if curtailed would result in suffering and death. There had of late years undoubtedly been a great increase in the cost of nursing and expenses connected therewith. There were many ways in which if they could afford it they ought to spend more, for what they were spending had produced admirable results. They had in hand large sums given by the public of the present day for the needs of the present day, and he was quite sure, with a clear income of £15,000 from investments, they ought not to fall behind other institutions in efficiency. As to the alterations to which Mr. Shaw Stewart had referred they were obliged to adopt one of two alternatives—either to convert or rebuild. Unlike some other hospitals, they were cooped up by buildings all round them, and were obliged to transmogrify an old building erected 60 or 70 years ago. If it should happen that they did outrun the purse of public charity, he confessed his sympathies would go out to the public, for one result at least would be that under Government management they would very nearly double the expenses of the hospitals. The expenses of conversion were now coming to an end. Had they adopted the alternative of pulling down and rebuilding, the result might conceivably have given them a better hospital, but it was quite certain it would have cost much more.

The Chairman having expressed his agreement in the regret at the diminution in their subscriptions, which he did not think creditable to the wealthy neighbourhood in which they stood, the accounts were received and adopted, and the other items on the agenda paper having been run through the meeting terminated.

### FRENCH HOSPITAL.

The annual general meeting of the French Hospital was held at the institution, Shaftesbury Avenue, on the 12th inst., Dr. Vintras taking the chair. The financial statement presented showed that the ordinary income for 1899 amounted to £4,163, the ordinary expenditure being £3,823. The extraordinary receipts (legacies) were £2,144, while the extraordinary expenses were £40. The debt on account of the building fund, which in 1898 was £4,829, had increased to £5,409. The number of in-patients treated during the year was 871, an increase of 107. The out-patients numbered 4,704, and the visits paid 14,974; 59 convalescents were received at the new home at Brighton during 1899, and there were now nine old people pensioners in residence there. The absence of Sir Wm. McCormac in South Africa was referred to, as was also that of Mr. Robert O'Callagan, surgeon and gynæcologist.

